# Advances in single-cell sequencing: insights from organ transplantation

**DOI:** 10.1186/s40779-021-00336-1

**Published:** 2021-08-13

**Authors:** Ying Wang, Jian-Ye Wang, Angelika Schnieke, Konrad Fischer

**Affiliations:** 1 Chair of Livestock Biotechnology, School of Life Sciences Weihenstephan, Technical University of Munich, Liesel Beckman Str 1, 85354 Freising, Germany; 2 Department of Surgery, School of Medicine, Technical University of Munich, 81675 Munich, Germany

**Keywords:** Single-cell RNA sequencing, Transplant rejection, Immune cell interactions, Transcriptional profiling, 10× Genomics chromium

## Abstract

Single-cell RNA sequencing (scRNA-seq) is a comprehensive technical tool to analyze intracellular and intercellular interaction data by whole transcriptional profile analysis. Here, we describe the application in biomedical research, focusing on the immune system during organ transplantation and rejection. Unlike conventional transcriptome analysis, this method provides a full map of multiple cell populations in one specific tissue and presents a dynamic and transient unbiased method to explore the progression of allograft dysfunction, starting from the stress response to final graft failure. This promising sequencing technology remarkably improves individualized organ rejection treatment by identifying decisive cellular subgroups and cell-specific interactions.

## Background

The demand for organs due to end-stage organ failure is permanently increasing. The best solution is autologous transplantation, enabled by patient-derived induced pluripotent stem cells. This approach is currently envisaged for single cells and basic cellular units such as islets. Tissue engineering is being optimized as one solution for larger organs. However, this option is not realistic presently. Xenotransplantation could soon become available clinically and an alternative backup for allotransplantation. However, both will depend on matching grafts and require immune suppression. The importance of understanding immune reactions, identifying cellular subtypes involved in graft acceptance and tolerance induction and identifying early indicators for rejection mechanisms require detailed immune cell profiling.

The immune system is a complex biological network comprising multiple layers of orchestrated genes, proteins and cells. In response to the challenge of pathogens or transplants, the immune system triggers various interactions between immune cells and other cells, provoking specific responses [1]. Innate and adaptive immune cells interact to ensure tissue protection according to functional requirements. Disruption of immune system homeostasis causes transplant rejection [2, 3]. Although inhibition of acute rejection has improved significantly in the past two decades, long-term rejection and immunosuppression can lead to high morbidity and mortality, and chronic transplant rejection can cause irreversible damage to the graft [4, 5]. The most common clinical acute rejection is mainly mediated by cellular responses, while hyperacute rejection and chronic rejection are mainly mediated by humoral immunity. The transplant rejection mechanism is an immunological reaction that recognizes foreign molecules of the donor cells, triggering attack and destruction. Various immunological factors are involved in acute and chronic rejection, including human leukocyte antigen (HLA) mismatch, donor-specific antibodies and non-immune factors (e.g., donor age, infection, and immunosuppressive drug toxicity) [6, 7].

## Assessing the immune reaction

Cellular and molecular assays to measure immune cell differentiation, cellular function and antigen specificity contribute to solving important problems in immune-mediated transplant rejection. Fluorescence-activated cell sorting (FACS) is used for post-transplantation immune surveillance to identify cells of the immune system by detecting surface marker expression or intracellular proteins, including cytokines. This tool has been supplemented by in situ histological tests [8, 9]. However, histological diagnosis may miss subtle alterations in individual patients, which are essential for rejective pathology [10]. Therefore, the combination of transcript sets and histological diagnosis of tissue samples is used to predict antibody-mediated or cellular rejections [11, 12]. Additionally, bulk RNA sequencing of allograft biopsies is a method to determine gene-specific single-nucleotide variants of donors and recipients [13]. Microarray technology has been used to study the pathogenesis of transplant rejection processes. For example, Roedder et al. [14] used microarray technology to determine a test set of 17 relevant genes to predict renal rejection. Each of these approaches provides valuable insights. However, they are subject to limitations due to the complex immune rejection response. Furthermore, extensive analysis cannot resolve phenotypic heterogeneity and distinguish the gene profile of donors and recipients in mixed cell populations [15]. Even with the application of Cytometry by Time of Flight (CyTOF), it remains challenging to assess a truly unbiased dataset of a single cell, a process that requires single-cell resolution [16]. Among single-cell profiling methods, scRNA-seq comprehensively measures the transcriptional expression of bulk cells [1, 17] and quantitatively analyzes all transcripts expressed in a single cell, providing an unbiased strategy to identify and characterize different cell populations [18–20].

This review discusses the development and application of scRNA-seq in organ transplantation. This cutting-edge technology will improve immunotherapies and help to predict recipient outcomes.

### Sample harvest and tissue processing

**Fig. 1:**
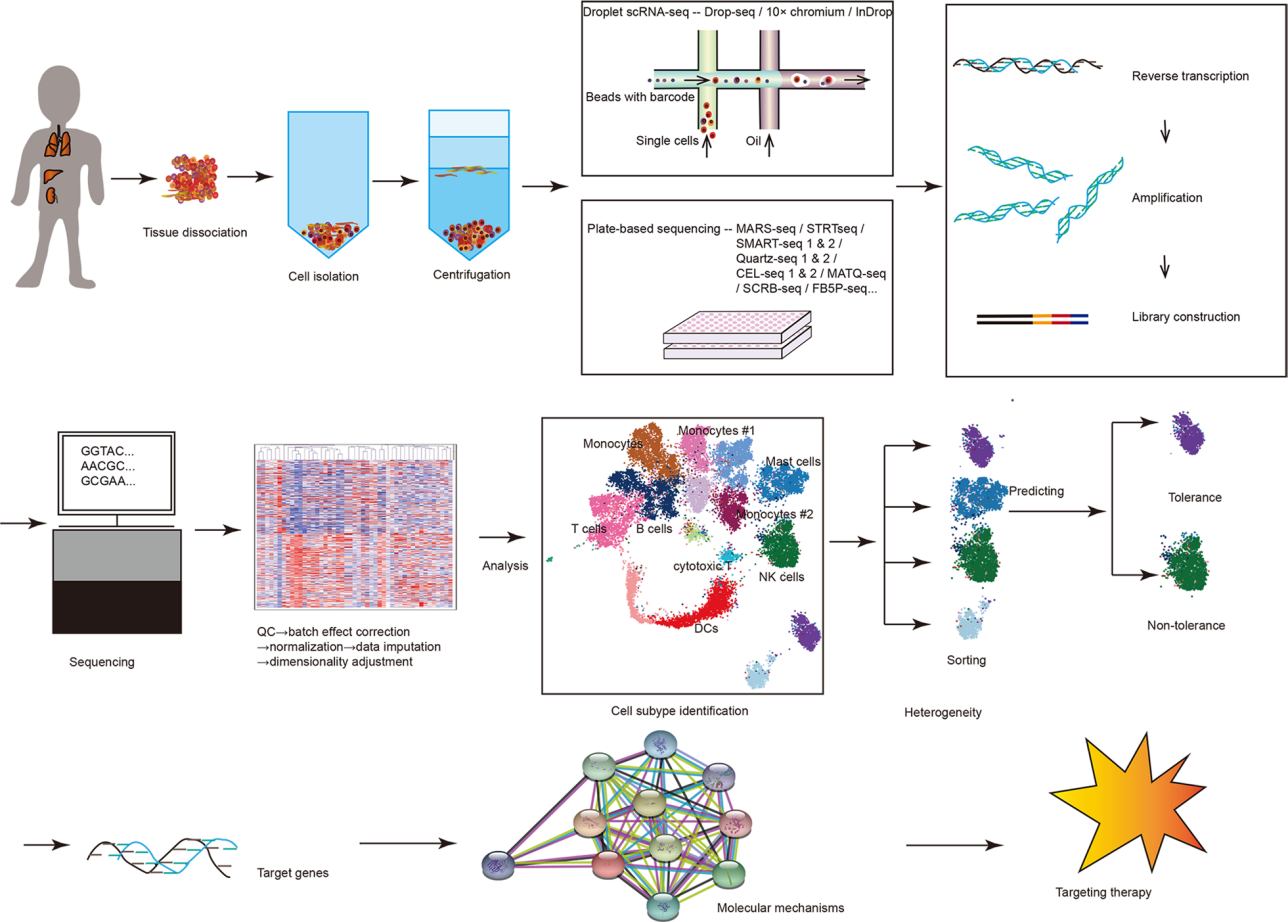
Steps of scRNA-seq to analyzing organ rejection. After biopsies of graft tissues, cells are isolated and can be used for droplet- or plate-based sequencing approaches. After batch effect correction, normalization, data imputation and dimensionality adjustments, specific cellular subtypes can be identified. Analysis of heterogeneity and prediction of tolerance is used to identify target genes and molecular interactions that are the basis for gene therapy approaches and long-term graft acceptance. QC quality control; DCs dendritic cells

The entire process starting with sample isolation to the final evaluation of scRNA-seq data is summarized in Fig. 1. The first step of scRNA-seq analysis is the dissociation of the graft tissue, which is obtained in most cases by biopsies. The currently used protocols were developed and improved over decades, and each has its strengths and weaknesses. For transplanted organs, laser capture microdissection, digestion or enzyme-related approaches, followed by density gradient centrifugation or fluorescence-activated cell sorting are used. Tissue dissociation is more difficult to implement for frozen samples, e.g., those of the liver [21]. In particular, for liver samples, a different sensitivity to cell death caused by the dissociation step may result in bias because hepatocytes die very fast, while other cells become activated during tissue dissociation, indicating a transcriptional stress response. Macparland et al. [22] developed a milder approach to reduce the cell damage rates by maintaining the tissue at 4 °C during all steps, including collagenase perfusion. Wang et al. [23] used a hypothermic strategy for kidney preservation for up to 4 days for scRNA-seq analysis. Recently, Guillaumet-Adkins et al. [24] published an improved method for sample preservation, gradual freezing by 1 ℃ per minute to -80 ℃, that does not change the transcriptional profiles and makes cryopreserved cells and tissues applicable for scRNA-seq. However, frozen tissues cannot be processed like fresh tissues. Tissue dissociation leads to the loss of spatial and anatomical information for cells, and the precise location of each cell should not be ignored. To address this issue, RNA probes identifying cellular transcriptional organization [25] or spatial transcriptomic protocols [26] are both helpful alternative methods.

### Single-cell methods

The next steps after tissue dissociation include RNA capture, reverse transcription, RNA sequencing, and library construction. Selection of a suitable sequencing method is challenging because several methods exist, such as CEL-seq2, Drop-seq, MARS-seq, MATQ-seq, Quartz-seq, SCRB-seq, Smart-seq, Smart-seq2, Drop-seq, FB5P-seq, SPLIT-seq, and DNBelab C4 [27–30]. We summarize the most commonly used scRNA-seq methods in **Table ****1** according to their capturing format, cDNA amplification, sequencing method, transcript coverage advantages and limitations. Microfluidic technologies for scRNA-seq involve droplet-based and plate-based technologies. Amplification is performed by PCR for Smart-seq [31, 32] and Quartz-seq [33, 34] and in vitro transcription, generating RNA in vitro, e.g., by InDrop [35] and CEL-seq [36, 37]. Drop-seq [38], InDrop [35], and CEL-seq [36, 37] incorporate unique molecular identifiers (UMIs) into cDNA. A UMI is a short sequence barcode to detect and quantify transcripts. These molecular barcodes uniquely tag each molecule in a sample library and reduce quantitative and error biases introduced by amplification. Smart-seq2, Quartz-seq, and MATQ-seq produce almost full-length sequencing data, while others (e.g., CEL-seq and Drop-seq) only capture the 3'-end sequence or 5'-sequence (e.g., FB5P-seq and STRT-seq) [10, 30, 39, 40]. Each platform provides multiple and specific but not completely comprehensive advantages in data capture. The reduction of mRNA amplification noise by CEL-seq2, InDrop, Drop-seq, MARS-seq, and SCRB-seq is a favorable feature that makes these platforms preferable. However, MARS-seq, SCRB-seq, and particularly Smart-seq2 platforms capture more genes using the same number of cells, making them preferable for relatively low quantities [41]. Drop-seq analyzes thousands of individual cells simultaneously without losing the original transcript [38]. Compared with other widely used single-cell RNA sequencing platforms (such as Smart-seq2), 10× Genomics Chromium is a more cost-effective and time-efficient system. This platform generates droplets and forms a single-cell suspension. Additionally, it can process many cells and detect even rare cell types or transcripts [42] by combining one of the following methods: InDrop for rare cell populations [38] or CEL-seq for complex tissues containing multiple cell populations [36, 37]. Smart-seq increases the thermal stability of DNA base pairs [31, 32]. MATQ-seq is implemented on low-abundance genes and noncoding and non-polyadenylated RNA [43]. Quartz-seq is applied to detect the different cell cycle phases and transcriptome heterogeneity. SCRB-seq is used for heterogeneous populations [33, 34]. FB5P-seq [30] and T-cell receptor repertoire sequencing (TCR-seq) [44] are used to identify the repertoire and diversity of BCRs and TCRs. STRT-seq tracks the cell origin efficiency without quantitative bias against long transcripts. One of the common disadvantages is the limited throughput and read coverage, e.g., by Smart-seq 1 and 2 [31, 32]. Another is the requirement for skilled technicians, e.g., for Quartz-seq 1 and 2 [33, 34]. Because each method has weaknesses (Table 1), investigators must choose a platform according to their specific interests.

**Table 1:** Comparison of scRNA methods

Method	Capture format	cDNA amplification	Transcript coverage	Molecule identifier	Strength	Weakness	References
Drop-seq	Droplets	PCR	3′ end	UMI	For individual cells; simultaneous analysis without losing the original transcript; high throughput, lower costs and reduction of the mRNA amplification noise	Only 3′ end is enabled for specific the amplification	[38]
10× Genomics Chromium	Droplets	PCR	3′ end	UMI	Increased throughput, cost effective and time efficient	Only 3′ end is enabled for specific amplification, efficiency losses	[45]
InDrop	Droplets	IVT	3′ end	UMI	Detects rare cell populations; high throughput, low noise profile	Low cell capture efficiency	[35]
CEL-seq 1 & 2	Plate	IVT	3′ end	UMI	For complex tissues containing multiple cell populations; improved accuracy and higher sensitivity; reduces mRNA amplification noise	Low cell capture efficiency	[36, 37]
Smart-seq 1 & 2	Plate	PCR	Full length	UMI	Increases thermal stability of DNA base pairs	Lower detection efficiency, limited throughput and read coverage	[31, 32]
MATQ-seq	Plate	PCR	Full length	NA	For low-abundance genes and noncoding and non-polyadenylated RNA; highly sensitive with quantitative detection efficiency	Low throughput	[43]
Quartz-seq 1 & 2	Plate	PCR	Full length	NA (Quartz-seq), UMI (Quartz-seq 2)	For different cell cycle phases and transcriptome heterogeneity detection; high quantity and efficiency in limited sequence reads	Requires skilled technician	[33, 34]
SCRB-seq	Plate	PCR	3′ end	UMI	For heterogeneous population identification; high throughput, low cost, simple steps, fewer potential biases, reduced mRNA amplification noise	Requires skilled technician	[46]
FB5P-seq	Plate	PCR	5′ end	UMI	For BCR and TCR repertoire identification; cost and time effective; integrative analysis of transcriptome	3′ end scRNA-seq protocols are not suitable	[30]
STRT-seq	Plate	PCR	5′ end	UMI	Improves efficiency and tracking of cell origin; no quantitative bias against long transcripts	Technical variation	[47]
TCR-seq	Plate	PCR	3′ end	NA	For T-cell diversity identification	No standardized thresholds, disparities between different studies	[44]
MARS-seq 1 & 2	Plate	IVT	3′ end	UMI	For in vivo transcriptional states in thousands of single cells; minimizes amplification bias	Requires skilled technician	[48]

*IVT* In vitro transcription, *UMI* Unique molecular identifiers

### Data analysis

Data analysis after sequencing generally comprises quality control (QC), batch effect correction, normalization, data imputation, dimensionality adjustment, subsequent expression analysis and cell subpopulation identification [39]. QC is required for high technical noise and low-quality data, which are often generated because of dead cells. Batch effects are caused by large-scale scRNA-seq datasets, samples prepared in different laboratories, even those based on the same protocol, and large data generated from separate technicians or different time points [49, 50]. They can cause systematic errors and differences among multiple transcriptional profiles. Thus, batch effect correction is vital to avoid this misinterpretation, and removing unwanted variation (RUV) is a good normalization strategy to adjust confounding technical effects [51]. Data normalization is necessary to adjust technical biases. Two types of normalization are used: sample normalization, which adjusts within-sample differences, and gene profile normalization, which eliminates gene expression biases. Data imputation is an effective strategy to insert substituted values into dropouts, eliminating the influence of missing data. Because of technological developments in scRNA-seq and bulk data generation with high dimensionalities, computational bioinformatics analysis is essential to process raw data. Automatic annotation methods such as the "SingleR" package [52], online databases [53] and gene expression markers [54] can be used for cell marker identification. The commonly used "SingleR" labels new cells from the scRNA-seq dataset based on similarity to the reference dataset of samples with known labels.

Challenges during data analysis involve multiple biases in the entire procedure and high dimensional datasets [55]. The possibility of low-quality data or dropouts in the scRNA-seq results, caused by low viability or dead cells, can lead to misinterpretation [56]. An optimal method can avoid false results to enable correct transcriptional interpretation (Table 2). "Seurat" is an R package designed for QC, analysis and exploration of scRNA-seq data to reduce some biases [54, 57]. To distinguish technical biases from biological signals, Bayesian Inference for Single-cell ClUstering and ImpuTing (BISCUIT) provides a discernible advantage for graphical algorithms. BISCUIT imputes dropouts and improves both clustering and normalization [58]. Using these tools, the data are clustered to reduce technical variations (amplification bias, sequencing depth, GC content, capture inefficiency, and RNA content variations [59]). Because scRNA-seq data contain many genes and cells in high dimensions, large-scale computational resources are required. T-distributed stochastic neighbor embedding (tSNE) is an approach for high-dimensional data because of the computation time and potentially manifold embeddings for the same datasets from run to run [60]. PhenoGraph is also an algorithmic method used for partitions of high-dimensional data into distinct subgroups within complex tissues [61]. Dimensional reduction and uniform manifold approximation and projection (UMAP) are alternative methods for data analysis developed to project the data into lower dimensions and visualize cell clusters with high reproducibility without losing the properties of the original data [62]. Xu et al. described a new clustering algorithm of graph-based shared nearest neighbor (SNN)-Cliq implemented in Python and MATLAB software, considering the surrounding data points, including low-density region data and detecting more cell subtypes with high accuracy [63]. For zero-inflated data comprising an excessive number of zeros, zero inflated factor analysis (ZIFA) is a novel approach to reduce dataset dimensions [64]. "Harmony" is another R package with an efficient batch algorithm method to integrate multiple datasets and requires fewer computational resources. Korsunsky et al. [65] developed this method (https://github.com/immunogenomics/harmony) and applied it to large datasets integrated with spatial transcriptomics data.

**Table 2:** Tools for scRNA-seq data analysis

Challenge	Method	References
Multiple biases	"Seurat" is an R package designed for quality control, analysis, and exploration of scRNA-seq data	[54, 57]
	BISCUIT provides the graphical algorithm and imputes dropouts, improving both clustering and normalization and reducing the technical biases from biological signals	[58]
Dimension	tSNE, PhenoGraph and ZIFA are used for high dimensional data. UMAP and SNN project the data into lower dimensions	[59–64]
	"Harmony" is an efficient batch algorithm method to integrate multiple datasets and requires fewer computational resources	[65]

However, these algorithms are most commonly used for static analysis. Another promising means, considering a continuous transition between different cellular states, are the machine learning approaches listed in Table 3, Monocle [66], Monocle2 [67], TSCAN [68], Wishbone [69], Slingshot [70], Diffusion pseudotime software [71] and Wanderlust [72] allow the construction of trajectories of cells in dynamic gene regulation and explain normal physiological and pathophysiologic alterations of cellular subgroups in specific locations of the human body. Saelens et al. [73] concluded that Slingshot, TSCAN and Monocle2 exhibited better trajectory identification. Therefore, the combination of personalized medicine and artificial intelligence will become applicable in the near future [41]. This combination will provide a map of cells, considering temporal dynamics and spatial positioning by evaluating the pathological microenvironment, phase of the cell cycle and responses to clinical medication. For transplanted organs, selecting an optimized process for accurate subsequent analysis is highly recommended.

**Table 3:** Tools for scRNA-seq trajectory inference

Method	URL	References
Monocle	http://cole-trapnell-lab.github.io/monocle-release/	[66]
Monocle2	http://cole-trapnell-lab.github.io/monocle-release/	[67]
TSCAN	https://github.com/zji90/TSCAN	[68]
Wishbone	https://github.com/ManuSetty/wishbone	[69]
Slingshot	https://github.com/kstreet13/slingshot	[70]
Diffusion Pseudotime Software	https://static-content.springer.com/esm/art%3A10.1038%2Fnmeth.3971/MediaObjects/41592_2016_BFnmeth3971_MOESM375_ESM.zip	[71]
Wanderlust	https://github.com/wanderlust/wanderlust	[72]

## Applications of scRNA-seq for transplantation

Currently, the main difficulties for successful transplantation comprise, among others, best-matched donor selection and a reduction in lifelong immunosuppression [74]. For transplant rejection, the latest advances in scRNA-seq provide an opportunity to fully reveal new cell types and states without result bias and RNA degradation [75]. Snapshots of the single-cell transcriptome exhibit various stages of immune differentiation and activation, while these stages are rarely synchronized among cells [76]. At single-cell resolution, it can describe immune cells, stromal cells and new cell subtypes that undergo immune rejection and further compare the unique characteristics of the signaling pathways between different cellular subgroups [57]. Here, we describe the advantages of scRNA-seq in the fields of kidney, liver, lung and hematopoietic stem cell (HSC) transplantation and for immunological applications.

### Kidney transplantation

T cells play a crucial role in graft rejection. Most studies have focused on bulk methods based on biopsy samples without providing information about αβ chain pairing of T-cell receptors (TCRs) [77]. This lack can underestimate actual library differences and fails to reflect that T cells with the same TCR can exert opposite biological functions. ScRNA-seq technology overcomes the abovementioned limitations and brings library analysis a higher diversity [78]. ScRNA-seq detects T-cell subclones [79]. For example, Morris et al. [80] monitored donor-reactive T cells in patients with tolerant and non-tolerant kidney transplantation. Donor reactivity has been detected in patients with tolerance. The decrease in T-cell clones in non-tolerant patients did not show a reduced number of donor-reactive T cells. Contrary to data from tolerant patients undergoing standard immunosuppression, an expansion of donor-reactive T-cell clones was observed in peripheral blood [81–83]. Antibody-mediated rejection (AMR) in the kidney is not easily identified by histological diagnoses. However, a transcriptional expression profile can strengthen the diagnosis. AMR injury is the most common driver of late allograft loss [12, 84]. Several groups have performed scRNA-seq of kidney allograft undergoing AMR, where monocytes, B cells, plasma cells and T cells invade into the tissue [85] and donor endothelial cells (ECs) are the primary targets of the recipients’ humoral immune response [86, 87]. Thus, scRNA-seq provides a comprehensive understanding of subtle mechanisms in conceptualizing heterogeneous kidney rejection. Macrophages and T cells, activated in the recipient, significantly differ from the original populations in either the donor or recipient. Some of these immune cells exist for only a few days after transplantation, but macrophages can persist for several years [88]. Furthermore, immune cell populations residing in donor-derived tissues can be replaced by recipient cells, particularly during rejection [89]. Liu et al. [57] presented a novel heterogeneous profile of immune cells based on allograft biopsies and matching healthy kidneys by scRNA-seq, integrating the key alterations of molecular functions, establishing therapeutic surveillance for kidney allograft rejection and improving allograft survival [90]. They identified subclusters of cytotoxic T lymphocytes that exhibit a more proinflammatory role in renal allograft rejection, while activated B cells interacted with surrounding stromal cells, mostly emerging in allograft kidneys, leading to immune cell recruitment and an activated inflammatory response. Non-invasive urine or blood biomarkers are applicable for a limited group of pathologies [91], whereas invasive biopsies are used to profile non-circulating immune cells in transplant rejection, providing more diagnostic accuracy and prognostic biomarkers amenable to therapeutic tools [10, 85].

### Liver transplantation

The interaction of immune cells and liver cells in a transplant setting is a key mechanism for liver tolerance induction [92]. ScRNA-seq can improve the hepatic immune cellular map in interpreting the specific CD4^+^ T-cell subgroup from other T cells in liver transplant rejection and tolerance [93]. Immune cells such as dendritic cells (DCs), T cells, and probably NK cells interact with liver sinusoidal ECs and hepatocytes, adding specific signaling molecules to generate a tolerogenic state. Apoptosis of infiltrating T cells may be critical for allograft tolerance [93, 94]. Despite possible tolerance induction after liver transplantation, human liver allografts are likely to be rejected without applying immunosuppressive drugs [95]. Approximately 10%-30% of allograft recipients are diagnosed with acute cellular rejection [96], likely due to T-cell-related immune responses [97]. Applying scRNA-seq may better elucidate the cellular immune responses in the liver allograft.

### Lung transplantation

Using scRNA-seq to test single cells isolated from lung transplantation donors and lung fibrosis has revealed transcriptionally distinct populations of alveolar macrophages that express profibrotic genes in patients with pulmonary fibrosis [98, 99]. Some mesenchymal cell markers that play a role in Wnt/β-catenin signaling during lung regeneration and some previously described rare cell populations have been identified. These technologies can improve the diagnosis of patients with fibrosis after lung transplantation and can be used to identify patients most likely to benefit from targeted therapy and monitor their response during disease progression [100]. Mould et al. [101] assessed the cell populations between healthy samples and found highly conserved cellular heterogeneity in bronchoalveolar lavage (BAL) cells. By dynamically comparing the lungs of donors and recipients, persistent donor resident memory T cells are correlated with better clinical outcomes [102], providing a potential therapeutic tool for extended allograft survival.

### HSC transplantation

To study the rejection of transplanted HSCs, Dong et al. [103] used scRNA-seq to obtain a transcriptome-based classification of 28 hematopoietic cell types. According to this classification, most transplanted HSCs are dedicated to transcriptional immunophenotypical multipotent progenitors (tMPP1). Transcriptional analysis and functional evaluation showed that the proliferation of transplanted cells is accompanied by a gradually decreased percentage of HSCs [104]. However, a balance between proliferation, differentiation and stem cell maintenance likely exists. Graft-versus-host disease (GVHD) is the main complication of allogeneic hematopoietic cell transplantation (HCT) [105]. Acute GVHD is mediated by donor T cells [106]. TCR-seq, as a type of scRNA-seq, can clarify how acute GVHD occurs. Although donor T-cell pools have highly similar TCRs, the TCR repertoire after HCT is very specific to recipients. TCR recombination is highly stochastic and may not depend on evaluating the most expanded TCR clones in any single recipient but on the complex polyclonal architecture. These results can be used to guide clinical decisions to prevent or treat acute GVHD [78]. By analyzing skin and peripheral blood T cells using TCR-seq, host skin-resident T cells were found to have an unanticipatedly pathogenic impact on GVHD [107].

### Immunological applications of scRNA-seq

ScRNA-seq identifies gene profiles of various cell populations. This technical tool avoids the weakness of bulk analysis, which is likely to miss cell-specific signatures [85]. It also improves the understanding of immune cell ontogeny and interaction with stromal cells in a given organ. The advantages of scRNA are maximized by its combination with databases or improvements by other techniques [such as single-nucleus RNA sequencing (snRNA-seq)] [108]. The Immune Cell Atlas (ICA) is an essential part of the international Human Cell Atlas initiative (https://www.humancellatlas.org/), which collects samples from patients who have undergone rejection response and evaluates different reaction stages using scRNA-seq technology. By visualizing the dynamic observation of cell processes, the subtle transcriptional differences among cell types can be qualitative. The rejection response, caused by specific gene regulation, provides robust target genes and molecular mechanisms to diagnose and treat transplant rejection and identify potential diagnostic markers [109, 110]. The limitations of scRNA-seq due to fresh tissue requirements, artifactual transcriptional biases and loss of fragile or low viability cells can be overcome by snRNA-seq, enabling the storage of frozen samples and analysis using a quality comparable to scRNA-seq [108, 111–113]. The latest developments in snRNA-seq, imaging technologies [such as single-molecule fluorescence in situ hybridization (FISH)], proteomics (MIBI-TOF), and genomics all together provide benefits to further investigate cellular functions [114].

Classical immune cell characterization in different organs has limitations due to technologies such as microscopy and high-affinity antibody labeling. Additionally, conventional transcriptome studies may miss some essential immune cell subtypes [85]. ScRNA-seq is currently widely used in immunology to unravel the differentiation process of HSCs, resolve previously under recognized immune cellular heterogeneity, decipher the immune cellular repertoire and predict disease-related phenotypes [39, 115, 116]. ScRNA-seq can help identify HSC fate branch points during differentiation; for example, conventional dendritic cells (cDCs) rely on the abundance of Siglec‑H and Ly6C to determine the direction of cDC type 1 (cDC1) or cDC type 2 (cDC2) [117]. The myeloid progenitor cells that produce mast cells and eosinophils or monocytes and macrophages depend on the presence of GATA1 [118]. Jaitin et al. [119] performed scRNA-seq on DCs and found different states of bone marrow-derived and other immune tissue-derived cells. Using scRNA-seq, progenitor immune cells can be identified by analyzing transcriptional variations in immature bone marrow and mature resident immune cells of specific organs [22]. Many new types of immune cells were identified using scRNA-seq, which can analyze cell differentiation and cell lineages, including innate lymphocytes and lung interstitial macrophages [57]. Recent studies have shown the heterogeneity of hematopoietic progenitor cells with a mixed lymphoid phenotype using scRNA-seq. This technology can also be used to identify novel cell types in several diseases, for example, cancer diagnosis and efficacy evaluation. By analyzing circulating tumor cells (CTCs) from prostate cancer patients, Miyamoto et al. [120] found that CTCs are highly heterogeneous in gene expression. The special feature of CTCs is that they activate Wnt signaling, which increases resistance to drug therapy. By analyzing brain cells in Alzheimer’s disease, scRNA-seq also enables the identification of microglia and perivascular macrophages related to neurodegenerative diseases [121]. ScRNA-seq also improves the diagnosis of disease heterogeneity, such as identifying specific B-cell receptor signaling pathways and gene expression patterns in non-Hodgkin’s lymphoma. Most adult B-cell lymphomas exhibit a B-cell phenotype at the germinal center (GC). By combining scRNA-seq, the mixed characteristics of B cells derived from GC and follicular lymphoma (FL) revealed unique transcription characteristics [122, 123]. In the future, organ transplantation single-cell sequencing will likely help further elucidate the pathogenesis of transplant rejection and initiate the development of clinical trials and the emergence of more effective drugs to reduce the immune response associated with transplant rejection, thereby improving the quality of life of patients and extending patient survival.

## Conclusion

Taken together, scRNA-seq can accurately interpret gene expression data, recognize cell heterogeneity, including new cell types or subtypes, and take snapshots of gene expression during the transition of cells from one state to another. All data can be integrated to define the critical process of cell development and differentiation, reveal the key signaling pathways and understand the gene regulatory network that predicts immune function [124–127].

## Data Availability

Not applicable.

## Abbreviations

AMR: Antibody-mediated rejection
BISCUIT: Bayesian Inference for Single-cell ClUstering and ImpuTing
cDC: Conventional dendritic cell
CTC: Circulating tumor cell
CyTOF: Cytometry by Time of Flight
ECs: Endothelial cells
FACS: Fluorescence Activated Cell Sorting
FL: Follicular lymphoma
GC: Germinal center
GVHD: Graft-versus-host disease
HCT: Hematopoietic cell transplantation
HLA: Human leukocyte antigen
HSCs: Hematopoietic stem cells
ICA: Immune Cell Atlas
IVT: In vitro transcription
QC: Quality control
RUV: Remove unwanted variation
scRNA-seq: Single-cell RNA sequencing
SNN: Shared nearest neighbor
SnRNA-seq: Single-nucleus RNA sequencing
TCR-seq: T-cell receptor repertoire sequencing
tMPP1: Transcriptional immunophenotypical multipotent progenitors
tSNE: T-distributed stochastic neighbor embedding
UMAP: Uniform manifold approximation and projection
UMI: Unique molecular identifiers
ZIFA: Zero inflated factor analysis
